# Ohio State Dental Society

**Published:** 1886-01

**Authors:** E. G. Betty


### PROCEEDINGS.

### Proceedings and Discussions of the Ohio State Dental Society, Reorganized at Chillicothe, October 28th, 29th, and 30th, 1885.

REPORTED BY E. G. BETTY, D.D.S.

*Continued from December Number, page* 623.

### SECOND DAY—AFTERNOON SESSION.

The meeting was called to order at 2 o'clock by President James, when the minutes of the morning session were read and approved.

Dr. A. O. Rawls, of Lexington, Ky., read a supplement to his paper on Pyorrhœa Alveolaris, which was read at the Mississippi Valley Association of Dental Surgeons, in March last, and which appeard in the May number of the REGISTER. The paper is as follows:

